# Second victim experiences and perceived support among newly registered nurses following patient safety incidents: a cross-sectional study

**DOI:** 10.3389/fpubh.2026.1813803

**Published:** 2026-05-19

**Authors:** Nan Xie, Lei Ye, Ping Tan

**Affiliations:** 1 Emergency Department of West China Hospital, West China School of Nursing, Sichuan University, Chengdu, China; 2 Institute of Disaster Medicine, Sichuan University, Chengdu, China; 3 Outpatient Department, West China Hospital, Sichuan University, Chengdu, China; 4 Department of Urology, Institute of Urology, Sichuan Clinical Research Center for Urological and Kidney Diseases, West China Hospital, Sichuan University, Chengdu, China

**Keywords:** cross-sectional study, newly registered nurses, patient safety, post-traumatic stress disorder, professional identity, second victims

## Abstract

**Aims:**

This study aimed to explore the second victim experiences of newly registered nurses (NRNs) and assess the roles of professional identity and post-traumatic stress disorder (PTSD) symptoms in influencing their distress and perceived support.

**Methods:**

An online questionnaire was used to collect demographic information, Second Victim Experience and Support Tool scores, Professional Identity Scale scores, and PTSD Symptom Scale scores. Data were analyzed using independent *t*-tests, ANOVA, Pearson’s correlation, and multiple linear regression. A significance level of *p* < 0.05 was applied.

**Results:**

No significant differences were observed in total Chinese version of the Second Victim Experience and Support Tool (C-SVEST) scores across demographic or clinical variables. However, multiple factors were associated with specific dimensions of second victim experiences. Educational background, years of experience, type of incident, and perceived responsibility were all significantly associated with subscale-level variations in distress and perceived support. Professional identity was associated with lower levels of work-related distress and higher levels of perceived support, whereas PTSD symptoms were associated with increased distress and reduced support. Regression analyses indicated that professional identity predicted lower levels of practice distress and higher levels of non-work-related support, while PTSD symptoms were significant predictors of physical distress and reduced support.

**Conclusion:**

This study highlights the multidimensional nature of second victim experiences among newly registered nurses following patient safety incidents. PTSD symptoms were associated with increased distress and reduced perceived support, whereas professional identity was associated with lower levels of practice-related distress and higher levels of perceived non-work-related support. These findings highlight the need to strengthen trauma-informed organizational support and to foster professional identity, aiming to improve coping mechanisms and support for nurses who experience second victim events.

## 1 Background

Patient safety incidents (PSIs) refer to events that occur during medical care and result in unnecessary harm to patients (1). These incidents include, but are not limited to, adverse drug reactions, medication extravasation, transfusion reactions, patient falls, pressure injuries, and unplanned extubations (2, 3). However, patients are not the only victims of these events. Healthcare professionals involved in PSIs may also experience psychological and physical consequences, thus becoming what is known as “second victims.” (4) These individuals often experience significant emotional distress, including anxiety, depression, self-blame, and guilt, as well as PTSD-related symptoms such as sleep disturbances (e.g., insomnia) (5). These negative effects may persist throughout their professional careers, severely impacting both mental health and clinical performance (6).

The term ‘second victim’ was first introduced by Wu (7) to describe healthcare providers who are emotionally affected by adverse events. This concept was later expanded upon by Scott et al. (8). They further emphasized that the harm caused by PSIs extends beyond patients and can result in substantial psychological trauma for providers, thereby impairing their professional competence and wellbeing. The second victim phenomenon has been widely documented across global healthcare systems (9). Healthcare workers may go through six psychological stages after experiencing a PSI: initial chaos and response, intrusive reflections, restoration of personal integrity, endurance of the inquisition, receipt of emotional first aid, and transition to moving on. Without adequate support or timely interventions, these reactions may develop into burnout, a desire to leave the profession, and decreased quality of care and patient safety (10–13). The prevalence of second victim experiences has been reported across different healthcare systems worldwide, with studies suggesting that approximately 10 to 50% of healthcare professionals may experience significant emotional distress following patient safety incidents (14). Similar patterns have been observed in the United States, Europe, and Asian countries, although variations may exist due to differences in healthcare systems, reporting cultures, and support structures. Importantly, previous research has shown that second victim experiences may negatively influence the reporting of medical errors and near misses. Fear of blame, emotional distress, and lack of organizational support can discourage healthcare professionals from reporting incidents, thereby hindering efforts to improve patient safety (15).

Newly registered nurses (NRNs), generally defined as registered nurses with less than 2 years of clinical experience, are particularly vulnerable in this context. Due to their limited experience and underdeveloped clinical skills, they often feel nervous and insecure in complex and unpredictable environments (16). Their lower psychological resilience makes them more susceptible to second victim experiences when involved in PSIs. Previous studies have shown that healthcare workers involved in PSIs may suffer from emotional distress, including anxiety, depression, self-blame, and guilt, often accompanied by sleep disturbances (17). Compared to experienced doctors or senior nurses who tend to recover more quickly, students and less experienced nurses typically show higher levels of anxiety and self-reproach (18). However, there is limited research specifically addressing NRNs as second victims, particularly focusing on their emotional and psychological responses and coping mechanisms, which this study aims to investigate.

In China, tertiary hospitals are characterized by high patient volumes and heavy workloads, which have been associated with increased occupational stress among nurses (19, 20). In these high-intensity environments, NRNs may be particularly vulnerable due to their limited clinical experience and the challenges of ongoing role transition. This transition has been conceptualized as “transition shock,” involving role ambiguity, increased responsibility, and psychological stress (21). These factors may increase their vulnerability to second victim experiences following patient safety incidents.

This study aims to explore the characteristics of second victim experiences among NRNs, identify psychological and physiological responses following patient safety incidents, and examine factors associated with these experiences. A deeper understanding of these processes may inform the development of targeted support strategies, such as psychological support systems and structured training programs. Previous studies have emphasized that effective support for second victims is essential for improving healthcare professionals’ wellbeing and maintaining patient safety (6, 7).

## 2 Methods

### 2.1 Study design, population, and setting

This study adopted a cross-sectional survey design to explore the psychological and physical responses of NRNs as second victims after experiencing PSIs. The study population comprised NRNs with less than 2 years of clinical nursing experience at a tertiary teaching hospital in China.

The inclusion criteria were as follows: (1) possession of a registered nurse license and active engagement in frontline clinical nursing work, (2) ≤ 2 years of clinical experience, and (3) informed consent and voluntary participation.

The exclusion criteria included the following: (1) individuals experiencing psychological distress due to non-PSI-related factors (e.g., personal or family issues) and (2) those currently undergoing psychological therapy or taking psychiatric medications.

The study was conducted in a large tertiary teaching hospital in China, involving approximately 1,000 NRNs during the study period. NRNs typically undergo a structured orientation program that includes clinical training, patient safety education, and mentorship from senior staff. However, formal psychological support specifically addressing second victim experiences is not routinely provided.

### 2.2 Sample size and sampling

A convenience sampling method was employed, which may limit the generalizability of the findings. Future studies using random sampling across multiple institutions are recommended for more robust results. The minimum required sample size was calculated using OpenEpi version 3.01,^1^ based on a population size of approximately 1,000 NRNs at the study hospital, a 95% confidence level, and a 5% margin of error, resulting in a minimum sample size of 278 participants. A conservative inflation factor of approximately 30–40% was applied to account for potential non-response and incomplete questionnaires, consistent with established survey methodology recommendations (22, 23) and empirical evidence from nursing research indicating substantial variability in response rates, often ranging between 40 and 60% (24). Accordingly, 450 questionnaires were distributed to ensure that the final analyzable sample would exceed the required minimum threshold. Ultimately, 413 valid responses were collected, resulting in an effective response rate of 91.8%.

### 2.3 Data collection

Data were collected using a structured questionnaire consisting of the following three sections:

1. *Demographic information*: This section includes gender, age, education level, years of experience, and department type.
2. *Experience with patient safety incidents*: Participants were asked whether they had encountered any PSIs during their clinical work, the type of incident (e.g., adverse drug events, unplanned extubation, or patient falls), and its perceived impact.
3. Assessment of Second Victim Psychological and Physical Responses:

The Chinese version of the Second Victim Experience and Support Tool (C-SVEST) was utilized. This tool assesses psychological distress, physical distress, professional self-efficacy, colleague support, managerial support, and non-work-related support, comprising 24 items across 6 dimensions (25). Higher scores indicate greater levels of the corresponding constructs; no reverse scoring is applied, and the total score is calculated by summing all subscale scores, reflecting the overall burden of the second victim experience.

*PTSD Symptom Scale (PTSD-SS)*: This scale evaluates symptoms of post-traumatic stress disorder following a PSI. The PTSD-SS was administered to all participants to assess post-traumatic stress symptoms, irrespective of the severity of the PSI.

*Professional Identity Scale*: This scale measures the level of professional identity among NRNs, addressing aspects such as professional values, sense of belonging, professional beliefs, and job satisfaction.

### 2.4 Ethical considerations

This study was approved by the Ethics Committee of West China Hospital of Sichuan University (No. 2024/2058) and the Chinese Clinical Trial Registry (Approval No. ChiCTR2500096267). Informed consent was obtained from all participants prior to data collection. The researchers explained the study’s purpose, confidentiality principles, and anonymity measures. Data were used exclusively for academic research, and no personally identifiable information was disclosed or used for non-research purposes.

### 2.5 Data analysis

Data analysis was conducted using SPSS version 29.0. Normality of variables was assessed using the Kolmogorov–Smirnov test. Continuous variables conforming to normal distribution were expressed as mean ± standard deviation (Mean ± SD); non-normally distributed variables were presented as median (interquartile range, IQR). Group comparisons were conducted using independent samples *t*-tests (for normally distributed variables) and Mann–Whitney U-tests (for non-normally distributed variables). One-way ANOVA was used to compare three or more groups, and Levene’s test was performed to assess the homogeneity of variances when necessary. Categorical variables were not included in the main statistical analysis; thus, chi-squared tests were not performed. Depending on normality, Pearson’s or Spearman’s correlation analyses were performed to explore the associations between second victim experiences (C-SVEST scores), PTSD symptoms, and professional identity. To further investigate the impact of professional identity and PTSD symptom severity on different dimensions of the second victim experience, multiple linear regression analyses were conducted using the Enter method. The independent predictive effects on each subscale of the C-SVEST were examined. All statistical tests were two-tailed, with a *p*-value of < 0.05 considered statistically significant. Given the exploratory nature of this study, multiple statistical comparisons were conducted without formal correction, which may increase the risk of type I errors. Consequently, the findings should be interpreted with caution, particularly those at marginal levels of statistical significance.

## 3 Results

### 3.1 Demographics of study participants

A total of 413 NRNs were included in this study, with a mean age of 23.26 ± 1.85 years.

The majority of participants were women (72.2%), held an undergraduate degree (88.4%), were single (96.9%), and had 1 year of clinical experience (77.2%). No significant differences were observed in the total C-SVEST scores across demographic or clinical variables (all *p* > 0.05), although some of the comparisons approached significance. However, significant differences emerged at the subscale level. Female nurses reported higher levels of psychological distress (*p* = 0.008) and practice distress (*p* = 0.020), as well as greater perceived management and non-work-related (both *p* < 0.001). Education level was associated only with physical distress (*p* = 0.014), while marital status showed no significant associations. Work experience was associated with higher levels of physical distress (*p* = 0.014) and lower levels of perceived support from colleagues, management, and in non-work-related areas (all *p* < 0.001).

The number of patient safety incidents was not associated with total scores (*p* = 0.084); however, it was related to psychological distress (*p* = 0.041) and various support dimensions, including colleague support (*p* = 0.027), management support, and non-work-related support (both *p* < 0.001). The timing of the last incident was not associated with total scores (*p* = 0.389), but it differed across all subscales (all *p* < 0.001), with the highest levels of distress reported for incidents that occurred between 6 months and 1 year.

Incident type and severity were not associated with total scores (*p* = 0.892 and *p* = 0.806), but showed significant associations with several (but not all) distress and support subscales. Nurse responsibility was not associated with total scores (*p* = 0.300), but primary responsibility was linked to higher levels of distress across psychological, physical, and practice domains (all *p* < 0.05), as well as higher levels of perceived support across all support domains (all *p* < 0.001) (Table 1).

**Table 1:** Demographic and clinical characteristics of participants.

Variables	Number (%)	C-SVEST total score (Mean ± SD)	Psychological distress (Mean ± SD)	Physical distress (Mean ± SD)	Practice distress (Mean ± SD)	Colleague support (Mean ± SD)	Management support (Mean ± SD)	Non-work-related support (Mean ± SD)
Gender								
Men	115 (27.8)	85.35 ± 13.66	13.65 ± 2.18	8.11 ± 2.28	16.45 ± 2.94	9.82 ± 1.17	24.85 ± 2.32	6.49 ± 1.59
Women	298 (72.2)	82.82 ± 13.21	14.39 ± 3.32	8.56 ± 3.27	17.33 ± 3.60	10.11 ± 2.31	26.72 ± 4.02	8.01 ± 1.52
*p*-value		0.084	0.008	0.120	0.020	0.090	<0.001	<0.001
Education level								
Junior college	28 (6.8)	78.68 ± 8.48	14.43 ± 3.43	9.50 ± 3.24	18.14 ± 3.32	10.61 ± 2.18	26.79 ± 4.10	7.68 ± 1.72
Undergraduate	365 (88.4)	84.00 ± 13.87	14.10 ± 3.08	8.28 ± 3.00	16.94 ± 3.50	9.96 ± 2.07	26.17 ± 3.72	7.59 ± 1.69
Postgraduate and above	20 (4.8)	81.55 ± 6.73	15.35 ± 1.93	9.80 ± 2.78	18.25 ± 2.07	10.55 ± 1.64	25.95 ± 3.19	7.40 ± 1.47
*p*-value		0.101	0.189	0.014	0.062	0.140	0.670	0.848
Marital status								
single	400 (96.9)	83.54 ± 13.52	14.17 ± 3.09	8.43 ± 3.04	17.09 ± 3.50	10.04 ± 2.08	26.26 ± 3.74	7.60 ± 1.69
married	13 (3.1)	83.08 ± 7.78	14.69 ± 1.70	8.69 ± 2.66	16.92 ± 1.32	9.62 ± 1.45	24.38 ± 2.36	7.31 ± 1.18
*p*-value		0.902	0.545	0.755	0.862	0.463	0.073	0.545
Working experience								
1 year	319 (77.2)	82.85 ± 13.23	14.30 ± 3.20	8.24 ± 3.08	17.05 ± 3.88	10.31 ± 2.02	26.55 ± 4.03	7.90 ± 1.54
2 years	94 (22.8)	85.81 ± 13.67	13.79 ± 2.47	9.11 ± 2.76	17.22 ± 1.13	9.07 ± 1.92	25.01 ± 1.98	6.53 ± 1.71
*p*-value		0.060	0.150	0.014	0.474	<0.001	<0.001	<0.001
Number of patient safety incident experienced								
Once	348 (84.3)	83.07 ± 13.20	14.35 ± 3.10	8.35 ± 3.16	17.11 ± 3.69	10.15 ± 2.20	26.58 ± 3.77	7.93 ± 1.47
Twice	49 (11.8)	84.51 ± 13.13	13.43 ± 2.60	8.69 ± 2.02	16.59 ± 1.74	9.37 ± 0.93	23.90 ± 2.78	6.16 ± 1.56
Three times or more	16 (3.9)	90.44 ± 16.43	13.00 ± 3.10	9.5 ± 2.58	18.00 ± 0.00	9.5 ± 0.52	25 ± 2.07	4.5 ± 0.52
*p*-value		0.084	0.041	0.270	0.341	0.027	<0.001	<0.001
Time of the last patient safety incident								
< 1 week	22 (5.3)	87.45 ± 15.52	12.27 ± 1.28	6.32 ± 1.49	17.18 ± 0.85	6.32 ± 1.49	25.23 ± 1.07	8.05 ± 0.21
1 week–1 month	69 (16.7)	82.93 ± 12.66	11.96 ± 3.30	6.72 ± 0.95	15.45 ± 3.18	9.82 ± 0.38	25.30 ± 1.19	6.84 ± 2.39
1–6 month(s)	195 (47.3)	84.24 ± 13.25	14.20 ± 2.43	8.53 ± 2.98	16.81 ± 3.04	10.18 ± 1.76	25.88 ± 3.89	7.42 ± 1.55
6 months–1 year	95 (23.0)	82.25 ± 13.22	16.33 ± 2.77	10.00 ± 3.52	19.02 ± 3.78	10.96 ± 2.43	28.16 ± 4.22	8.43 ± 1.07
1–2 year(s)	32 (7.7)	81.56 ± 14.42	13.88 ± 3.37	8.31 ± 2.84	16.50 ± 4.00	9.31 ± 2.05	25.00 ± 3.85	7.38 ± 1.60
*p*-value		0.389	<0.001	<0.001	<0.001	<0.001	<0.001	<0.001
Type of the last patient safety incident experienced								
Adverse events	178 (43.1)	83.52 ± 14.01	14.80 ± 2.22	9.01 ± 2.92	17.69 ± 2.56	9.66 ± 2.05	26.05 ± 3.48	7.58 ± 1.50
Reportable circumstances	2 (0.5)	89.50 ± 9.19	16.00 ± 0.00	12.00 ± 0.00	17.00 ± 0.00	10.00 ± 0.00	23.00 ± 0.00	7.00 ± 0.00
Near misses	12 (2.9)	85.25 ± 18.36	10.83 ± 5.24	7.50 ± 2.68	15.33 ± 1.67	8.83 ± 1.40	25.67 ± 0.78	8.00 ± 0.00
No-harm incidents	221 (53.5)	83.38 ± 12.61	13.86 ± 3.34	7.99 ± 3.05	16.70 ± 4.03	10.39 ± 2.04	26.38 ± 4.00	7.57 ± 1.86
*p*-value		0.892	<0.001	0.002	0.009	<0.001	0.466	0.806
Severity of patient harm								
No harm	257 (62.2)	83.51 ± 14.00	13.59 ± 3.31	7.97 ± 3.00	16.81 ± 3.98	10.21 ± 1.89	26.50 ± 3.92	7.63 ± 1.78
Mild harm	112 (27.2)	83.00 ± 12.85	15.11 ± 2.61	8.94 ± 3.05	17.80 ± 2.44	9.88 ± 2.56	26.29 ± 3.68	7.92 ± 1.34
Moderate harm	8 (1.9)	87.38 ± 10.36	15.25 ± 0.46	8.63 ± 1.41	16.25 ± 2.31	10.13 ± 0.83	25.50 ± 1.07	8.00 ± 0.00
Severe harm	36 (8.7)	84.39 ± 10.97	15.33 ± 1.12	10.17 ± 2.65	17.00 ± 1.43	9.17 ± 1.36	24.00 ± 1.17	6.17 ± 1.36
*p*-value		0.806	<0.001	<0.001	0.073	0.031	0.002	<0.001
Nurse’s degree of responsibility for the incident								
Primary responsibility	52 (12.6)	82.52 ± 10.35	16.75 ± 2.20	10.25 ± 3.85	18.10 ± 3.40	11.27 ± 2.24	29.00 ± 3.73	8.60 ± 1.39
Secondary responsibility	233 (56.4)	82.92 ± 12.67	13.70 ± 3.37	8.23 ± 2.91	17.14 ± 4.05	10.10 ± 1.91	26.58 ± 3.69	7.13 ± 1.87
Equal responsibility	128 (31.0)	85.04 ± 15.50	14.02 ± 2.11	8.06 ± 2.60	16.59 ± 1.82	9.40 ± 2.02	24.39 ± 2.78	8.00 ± 1.00
*p*-value		0.300	<0.001	<0.001	0.027	<0.001	<0.001	<0.001

Some subgroup analyses included small sample sizes; therefore, the results should be interpreted cautiously.

### 3.2 Correlations between PTSD symptoms, professional identity, and C-SVEST dimensions

Correlation analysis revealed significant associations between PTSD-SS and most C-SVEST dimensions, as well as weaker but significant associations with professional identity.

PTSD-SS was positively correlated with psychological distress (*r* = 0.409, *p* < 0.01), physical distress (*r* = 0.647, *p* < 0.01), and practice distress (*r* = 0.355, *p* < 0.01). It also showed positive correlations with colleague support (*r* = 0.386, *p* < 0.01) and management support (*r* = 0.144, *p* < 0.01), but no significant correlation with non-work-related support (*r* = −0.033, *p* > 0.05).

Professional identity was negatively correlated with practice distress (*r* = −0.111, *p* < 0.05) and colleague support (*r* = −0.115, *p* < 0.05), although the correlations were weak. No significant correlations were found between professional identity and psychological distress, physical distress, management support, or non-work-related support (*all p* > 0.05) (Table 2).

**Table 2:** Correlation between PTSD-SS, professional identity, and C-SVEST dimensions.

Variables	Psychological distress	Physical distress	Practice distress	Colleague support	Management support	Non-work-related support
PTSD-SS	0.409**	0.647**	0.355**	0.386**	0.144**	−0.033
Professional identity	−0.051	−0.044	−0.111*	−0.115*	−0.047	0.049

**p < 0.01 and *p < 0.05.

### 3.3 Multiple linear regression analysis: professional identity predicting C-SVEST dimensions

Multiple linear regression analyses demonstrated that professional identity had differential predictive effects across C-SVEST dimensions (Table 3).

**Table 3:** Multiple linear regression analysis: professional identity predicting C-SVEST dimensions.

Variables	Psychological distress	Physical distress	Practice distress	Colleague support	Management support	Non-work-related support
*β*	0.012	0.090	−0.173	−0.163	0.033	0.149
*t*	0.133	1.231	−2.386	−2.121	0.440	2.239
*p*-value	0.895	0.219	0.017	0.035	0.660	0.026

Professional identity significantly negatively predicted practice distress (*β* = −0.173, *t* = −2.386, *p* = 0.017), indicating that a higher professional identity was associated with lower levels of perceived practice-related distress. Similarly, professional identity was a modest negative predictor of colleague support (*β* = −0.163, *t* = −2.121, *p* = 0.035).

Unexpectedly, professional identity positively predicted non-work-related support (*β* = 0.149, *t* = 2.239, *p* = 0.026), suggesting that nurses with strong professional identities may perceive or seek more support from outside the workplace.

However, no significant predictive effects were found for psychological distress (*β* = 0.012, *p* = 0.895), physical distress (*β* = 0.090, *p* = 0.219), or management support (*β* = 0.033, *p* = 0.660).

### 3.4 Multiple linear regression analysis: PTSD-SS predicting C-SVEST dimensions

Regression analyses showed that PTSD-SS significantly predicted multiple C-SVEST dimensions (Table 4). Physical distress: PTSD-SS was the strongest positive predictor (*β* = 0.609, *t* = 11.385, *p* < 0.001), indicating that more severe PTSD symptoms were associated with greater physical distress. Practice distress: Furthermore, significantly positively predicted by PTSD-SS (*β* = 0.174, *t* = 3.260, *p* = 0.001). Management support: PTSD-SS negatively predicted perceived management support (*β* = −0.143, *t* = −2.560, *p* = 0.011), suggesting that higher PTSD symptoms may be associated with reduced perceptions of managerial support. Non-work-related support: PTSD-SS showed the strongest negative predictive effect (*β* = −0.229, *t* = −4.675, *p* < 0.001), indicating less perceived support outside of work among those with more severe PTSD symptoms. Colleague support: Although a positive trend was observed (*β* = 0.095), it did not reach statistical significance (*t* = 1.690, *p* = 0.092). Psychological distress: PTSD-SS did not significantly predict psychological distress (*β* = 0.003, *t* = 0.041, *p* = 0.967).

**Table 4:** Multiple linear regression analysis: PTSD-SS predicting C-SVEST dimensions.

Variables	Psychological distress	Physical distress	Practice Distress	Colleague support	Management support	Non-work-related support
*β*	0.003	0.609	0.174	0.095	−0.143	−0.229
*t*	0.041	11.385	3.260	1.690	−2.560	−4.675
*p*-value	0.967	<0.001	0.001	0.092	0.011	<0.001

## 4 Discussion

### 4.1 Factors associated with second victim experiences among NRNs

In this study, no significant differences were observed in total C-SVEST scores across demographic or clinical variables. Instead, variations were primarily observed at the subscale level, suggesting that second victim experiences among NRNs are better understood as a multidimensional construct rather than a single overall burden.

NRNs reported notable levels of psychological and physical distress following patient safety incidents, although these effects were not reflected in overall scores. This pattern indicates that different aspects of the second victim experience may be differentially influenced by individual and clinical factors. This finding is consistent with previous literature indicating that early-career nurses, due to limited clinical experience and underdeveloped coping strategies, are more likely to experience intense emotional and somatic reactions following patient safety incidents (26–28).

Gender differences observed in this study are broadly consistent with previous findings in occupational stress and burnout research, where variations have been attributed to differences in emotional labor, coping strategies, and workplace expectations rather than inherent traits (29). However, in the present study, these differences were observed at the subscale level rather than in total C-SVEST scores, suggesting that gender may influence specific aspects of distress and support rather than the overall burden of second victim experiences.

Educational background showed limited influence on second victim experiences. Consistent with the results, education level was only associated with physical distress, with no significant differences observed in total scores or other dimensions. Nurses with higher educational attainment may experience stronger professional responsibilities and expectations, which have been linked to increased feelings of guilt and self-blame following clinical errors (6). However, direct empirical evidence linking educational level to emotional responses in second victim contexts remains limited and warrants further investigation.

Years of experience and workplace dynamics were also associated with second victim experiences. In line with our findings, years of experience were not associated with the overall C-SVEST scores but were related to specific dimensions, particularly perceived support. Nurses with 2 years of experience reported lower perceived support, which may reflect a structural reduction in formal support following the transition from supervised orientation to independent practice. This may be due to opposite directions or compensatory effects across subscales, which can obscure differences in total scores. Previous research has shown that early-career nurses often experience a decrease in perceived organizational support, which may increase vulnerability to stress and burnout (21, 30).

The type of PSIs and level of responsibility were also significantly associated with distress levels. However, consistent with the results, these factors were not associated with total C-SVEST scores but were significantly associated with multiple distress and support dimensions. Nurses involved in reportable or adverse events experienced more psychological and physical distress, with those bearing primary responsibilities showing the highest levels of distress and support needs. These findings are consistent with Scott’s Second Victim Model (8), which highlights the roles of perceived responsibility and event severity in shaping psychological outcomes. Additionally, the lower levels of distress observed shortly after the incident may reflect acute coping mechanisms, such as emotional numbing, followed by delayed distress as individuals cognitively process the event (31, 32). However, as individuals cognitively process the event over time, distress may intensify, consistent with models of delayed psychological response to trauma (32).

An interesting and somewhat counterintuitive finding was that nurses who bore primary responsibility for patient safety incidents reported both the highest levels of distress and the highest perceived support from colleagues and managers. Importantly, this pattern was observed at the dimensional level rather than in overall scores. This may reflect reactive mobilization of organizational and peer support in response to high-severity or high-visibility incidents, whereby individuals most directly involved receive more immediate emotional and professional support. Similar patterns have been reported in previous studies, suggesting that support systems are often activated post-hoc in response to critical incidents rather than being proactively available (6, 8).

### 4.2 Role of PTSD symptoms in second victim experiences

PTSD symptoms were strong predictors of multiple dimensions of second victim experience, particularly physical distress (*β* = 0.609) and practice-related distress (*β* = 0.174). These findings are consistent with previous studies showing that PTSD is associated with autonomic nervous system dysregulation and somatic symptoms such as headaches, fatigue, or physical discomfort (31, 32), highlighting the physical expression of psychological trauma.

Notably, PTSD symptoms negatively predicted perceived management and non-work-related support. This may be explained by the tendency of individuals with PTSD to experience social withdrawal, emotional numbing, and reduced trust in others, which can impair help-seeking behaviors and perceptions of available support (33, 34). These findings highlight the importance of trauma-informed leadership in proactively identifying affected individuals and offering tiered, context-sensitive support strategies.

### 4.3 Role of professional identity in the second victim experience

This study also points to professional identity as a potential protective factor in second victim experiences. Consistent with previous research (35), higher professional identity was associated with lower practice-related distress, suggesting that a strong sense of professional self may enhance emotional regulation and coping capacity in clinical settings. These findings suggest that efforts to enhance professional identity, such as mentorship and reflective practice, may facilitate more effective coping with the emotional impact of patient safety incidents among NRNs.

In addition, nurses with higher professional identity scores reported reduced reliance on colleagues’ support and greater use of non-work-related support, suggesting a broader, more autonomous coping orientation. However, professional identity did not significantly predict psychological or physical distress, suggesting that its protective effects may be more closely related to role-related functioning rather than core emotional responses. These findings align with theoretical perspectives that conceptualize professional identity as enhancing role clarity, commitment, and resilience, while having a more limited impact on trauma-related psychological symptoms (36–38).

### 4.4 Implications for nursing management and education

The findings of this study can be better understood in a broader international context. Previous research from different regions suggests that the prevalence of second victim experiences ranges from approximately 10 to 50% among healthcare professionals. For example, studies conducted in the United States have reported prevalence rates ranging from 10 to 43%, depending on clinical settings and measurement approaches (4), while European studies have documented comparable ranges with variations influenced by institutional support systems.

In Asian countries, studies from China and South Korea have reported similar levels of psychological distress following patient safety incidents, but with notable differences in reporting behaviors and support utilization (13, 17). These differences may be influenced by cultural norms, hierarchical structures, and variations in organizational safety culture.

In addition, the impact of second victim experiences extends beyond individual wellbeing. Emotional distress and fear of negative consequences may reduce the willingness of healthcare professionals to report medical errors (12, 15). Underreporting remains a persistent challenge worldwide and may limit opportunities for organizational learning and patient safety improvement. Therefore, establishing effective support systems for second victims is critical not only to supporting healthcare workers but also to fostering a transparent, learning-oriented safety culture.

## 5 Limitations

This study has several limitations. First, the sample was drawn from a single tertiary hospital using convenience sampling, which may limit the generalizability of the findings. Second, data were self-reported, which may introduce subjective bias, particularly when assessing psychological experiences, as participants may underreport or suppress emotional reactions. Third, the cross-sectional design precludes causal inferences. Fourth, this study did not include subgroup analyses by clinical department, which may influence distress levels, as nurses working in high-acuity settings (e.g., intensive care units or emergency departments) may experience different levels of psychological burden. Future research should consider longitudinal designs to explore changes in second victim experiences over time and to assess the effectiveness of interventions. In addition, qualitative methods could be used to gain deeper insights into NRNs’ personal experiences and support needs, providing a richer theoretical foundation for support system development.

## 6 Conclusion

This study highlights the multidimensional nature of second victim experiences among NRNs following patient safety incidents. PTSD symptoms not only intensified distress but also diminished perceived support, further exacerbating psychological isolation. Conversely, professional identity was associated with lower practice-related distress and higher perceived non-work-related support, especially in reducing practice-related distress and promoting the mobilization of external support.

The findings highlight the urgency of addressing second victim phenomena among NRNs. Nursing administrators should prioritize the establishment of trauma-informed support systems and implement early identification protocols to support NRNs facing second victim experiences. Tailored, context-sensitive interventions should be provided based on the severity of trauma symptoms. Strengthening professional identity and fostering a sense of belonging may be associated with improved coping and adaptation following adverse events.

Future nursing management and education strategies should integrate psychological support, professional identity development, and safety culture promotion to support the healthy development and long-term career sustainability of the new nursing workforce.

## Data Availability

The raw data supporting the conclusions of this article will be made available by the authors, without undue reservation.

## Footnotes

1
www.openepi.com
